# Supplementary material for: A Note on Exploratory Item Factor Analysis by Singular Value Decomposition
Source: Psychometrika. 2020 May 26;85(2):358–72. doi: 10.1007/s11336-020-09704-7 (PMC7385012; doi:10.1007/s11336-020-09704-7)
Supplement: Supplementary file 1 — Supplementary material 1 (pdf 247 KB) [file 11336_2020_9704_MOESM1_ESM.pdf]

Supplement to "A Note on Exploratory Item Factor Analysis by Singular Value Decomposition"

A Notations

Let $\Theta^* = (\boldsymbol{\theta}_1^*, ..., \boldsymbol{\theta}_N^*)^\top = (\theta_{ik})_{N\times K}, A^* = (\mathbf{a}_1^*, ..., \mathbf{a}_J^*)^\top = (a_{jk})_{J\times K}$, and $\mathbf{d}^* = (d_1^*, ..., d_J^*)$ denote the true person parameters, factor loadings and intercept parameters, respectively. We also denote $\boldsymbol{\theta}_i^+ = (1, (\boldsymbol{\theta}_i^*)^\top)^\top, \mathbf{a}_j^+ = (d_j^*, (\mathbf{a}_j^*)^\top)^\top, \quad$ for $i = 1, ..., N, \quad j = 1, ..., J$. We use $\mathbf{1}_N, \mathbf{0}_N$ to denote N dimensional vectors with all entries being 1 and 0 respectively, and $B_{\mathbf{a}}^{(K)}(C)$ to denote the ball in $\mathbb{R}^K$ centered at $\mathbf{a} \in \mathbb{R}^K$ with radius C. For a matrix $Z = (z_{ij})_{m\times n}$ and a function $f : \mathbb{R} \to \mathbb{R}$, let $f(Z) := (f(z_{ij}))_{m\times n}$. Let $\sigma_k(Z)$ denote the k-th largest singular value of Z, and $\|Z\|, \|Z\|_*$ denote the spectrum norm and nuclear norm of Z, which is the largest singular value and the sum of all singular values, respectively. If Z is a square matrix, let $\lambda_k(Z)$ denote the k-th largest eigenvalue of Z.

We denote

$$X^* := (x_{ij}^*)_{N\times J} = f(\Theta^*(A^*)^\top + \mathbf{1}_N(\mathbf{d}^*)^\top)$$

as the true probability matrix and define $\tilde{X} = (\tilde{x}_{ij})_{N\times J}$ by

$$\tilde{x}_{ij} = \begin{cases} 0, & \text{if } x_{ij} < 0, \\ x_{ij}, & \text{if } 0 \le x_{ij} \le 1, \\ 1, & \text{if } x_{ij} > 1, \end{cases}$$

where x_{ij} is defined in step 5 of Algorithm 2.

Throughout the proof, we use c to denote constant, whose value may change from line to line or even within a line. We will drop the subscripts in $\epsilon_{N,J}$ and write ϵ for notional simplicity.

B Proof of Theorems

Proof of Theorem 1. Since Theorem 1 is a special case of Proposition 4 when $p = 1$ and $W = \mathbf{1}_N \mathbf{1}_J^\top$, we refer the readers to the proof of Proposition 4. □

Proof of Theorem 2. Let σ_k^* denote the kth largest singular value of $\Theta^*(A^*)^\top$. Then we have

$$|\hat{\sigma}_k - \sigma_k^*| \le \|\hat{M} - \Theta^*(A^*)^\top\|_2 \le \|\hat{M} - \Theta^*(A^*)^\top\|_F. \tag{B.1}$$

By (D.12) in the proof of Lemma 1, we can get

$$\frac{1}{\sqrt{NJ}} \|\hat{M} - \Theta^*(A^*)^\top\|_F \overset{pr}{\to} 0. \tag{B.2}$$

Notice that (B.2) holds as long as the input dimension in the algorithm is fixed. Combine (B.1) and (B.2) to have

$$\frac{|\hat{\sigma}_k - \sigma_k^*|}{\sqrt{NJ}} \overset{pr}{\to} 0.$$

Notice that $\sigma_{K+1}^* = 0$ and we get

$$\frac{\hat{\sigma}_{K+1}}{\sqrt{NJ}} \overset{pr}{\to} 0.$$

For $k = K$, we get

$$\Pr\left(\frac{|\hat{\sigma}_K - \sigma_K^*|}{\sqrt{NJ}} \le \tilde{\epsilon}\right) \to 1$$

for any $\tilde{\epsilon} > 0$ and thus

$$\Pr\left(\frac{\hat{\sigma}_K}{\sqrt{NJ}} \ge \frac{1}{\sqrt{NJ}}\sigma_K^* - \tilde{\epsilon}\right) \to 1. \tag{B.3}$$

For σ_K^*, we have

$$\begin{aligned}\frac{1}{\sqrt{NJ}}\sigma_K^* &= \frac{1}{\sqrt{NJ}}\sigma_K(\Theta^*(A^*)^\top) \\ &\geq \frac{1}{\sqrt{N}}\sigma_K(\Theta^*)\frac{1}{\sqrt{J}}\sigma_K(A^*) \\ &\geq C_1\frac{1}{\sqrt{N}}\sigma_K(\Theta^*).\end{aligned} \tag{B.4}$$

The last inequality is due to condition A4. Let $\hat{\Sigma} = \frac{1}{N}\sum_{i=1}^N \boldsymbol{\theta}_i^*(\boldsymbol{\theta}_i^*)^\top$ and it is not hard to verify that

$$\frac{1}{\sqrt{N}}\sigma_K(\Theta^*) = \sqrt{\lambda_K(\hat{\Sigma})}.$$

By law of large number, we know

$$\|\hat{\Sigma} - \Sigma^*\|_2 \xrightarrow{pr} 0$$

which leads to

$$\lambda_K(\hat{\Sigma}) \xrightarrow{pr} \lambda_K(\Sigma^*) > 0$$

and thus

$$\frac{1}{\sqrt{N}}\sigma_K(\Theta^*) = \sqrt{\lambda_K(\hat{\Sigma})} \xrightarrow{pr} \sqrt{\lambda_K(\Sigma^*)} > 0. \tag{B.5}$$

Combining (B.3), (B.4), (B.5) and choosing $\tilde{\epsilon} = \frac{1}{2}\sqrt{\lambda_K(\Sigma^*)}$, we have

$$\Pr\left(\frac{\hat{\sigma}_K}{\sqrt{NJ}} > \frac{1}{4}\sqrt{\lambda_K(\Sigma^*)}\right) \to 1.$$

We complete the proof by choosing $\delta = \frac{1}{4}\sqrt{\lambda_K(\Sigma^*)}$. □

C Proof of Propositions

Proof of Proposition 1. According to the choice of ϵ, we have $h(2\epsilon) \geq C\sqrt{C_0^2+1}$. Then,

$$\Pr\left(\|\boldsymbol{\theta}_1^*\| \geq h(2\epsilon)/C\right) = 0, \text{ and } \frac{(h(2\epsilon_{N,J}))^{\frac{K+1}{K+3}}}{(\epsilon_{N,J}g(\epsilon_{N,J}))^2} = O(1).$$

We complete the proof by Theorem 1. □

Proof of Proposition 2. For the logistic link function, we have

$$h(y) = \log \frac{1-y}{y} \text{ and } g(y) = y(1-y), \quad \text{for } y \in (0, 0.5). \tag{C.1}$$

Since $\boldsymbol{\theta}_1^*$ is a sub-Gaussian random vector, then $\|\boldsymbol{\theta}_1^*\|_2^2$ is an sub-exponential random variable, which means there exist constant $c_1, c_2 > 0$, such that for any $t > 0$, we have

$$\Pr(\|\boldsymbol{\theta}_1^*\|^2 \geq t) \leq c_1 \exp(-c_2 t).$$

Then,

$$\begin{aligned} \Pr\left(\|\boldsymbol{\theta}_1^*\| \geq \frac{h(2\epsilon)}{C}\right) &= \Pr\left(\|\boldsymbol{\theta}_1^*\|^2 \geq \frac{(h(2\epsilon))^2}{C^2}\right) \\ &= \Pr\left(\|\boldsymbol{\theta}_1^*\|^2 \geq \frac{\log^2\left(\frac{1-2\epsilon}{2\epsilon}\right)}{C^2}\right) \\ &\leq c_1 \exp\left(-c_2 \frac{\log^2\left(\frac{1-2\epsilon}{2\epsilon}\right)}{C^2}\right) \end{aligned} \tag{C.2}$$

Recall we choose $\epsilon = \gamma_0 J^{-\gamma_1}$ in (9). Consequently,

$$\log^2\left(\frac{1-2\epsilon}{2\epsilon}\right) = \gamma_1^2 (\log J)^2 + O(\log J). \tag{C.3}$$

Therefore,

$$\begin{aligned} c_1 \exp\left(-c_2 \frac{\log^2\left(\frac{1-2\epsilon}{2\epsilon}\right)}{4C^2}\right) =& c_1 \exp\left(-c_2 \frac{\gamma_1^2(\log J)^2}{C^2} + O(\log J)\right) \\ \leq& c_1 \exp\left(-c_2 \frac{\gamma_1^2(\log N)^2}{C^2\beta^2} + O(\log N)\right) \\ =& o\left(\frac{1}{N}\right) \end{aligned} \tag{C.4}$$

where the second inequality is due to the assumption that $J^\beta \geq N$. The above display

together with (C.2) verifies (3). We proceed to verify (4). According to (C.1), we have

$$\frac{(h(2\epsilon))^{\frac{K+1}{K+3}}}{(\epsilon g(\epsilon))^2} = \frac{\left(\log\left(\frac{1-2\epsilon}{2\epsilon}\right)\right)^{\frac{K+1}{K+3}}}{\epsilon^4(1-\epsilon)^2}.$$

Plugging in $\epsilon = \gamma_0 J^{-\gamma_1}$, the above equation becomes

$$\frac{\left(\log\left(\frac{1-2\epsilon}{2\epsilon}\right)\right)^{\frac{K+1}{K+3}}}{\epsilon^4(1-\epsilon)^2} = (1+o(1))\,\gamma_0^{-4} J^{4\gamma_1} \left(\gamma_1 \log J + O(1)\right)^{\frac{K+1}{K+3}} = J^{4\gamma_1 + o(1)}.$$

Thus, for $\gamma_1 \in (0, \frac{1}{4(K+3)})$,

$$\frac{\left(\log\left(\frac{1-2\epsilon}{2\epsilon}\right)\right)^{\frac{K+1}{K+3}}}{\epsilon^4(1-\epsilon)^2} = o(J^{\frac{1}{K+3}}) \tag{C.5}$$

This verifies (4) and completes the proof by applying Theorem 1.

□

Proof of Proposition 3. The proof of Proposition 3 is similar to proof of Lemma 1. We will only state the main steps and omit the repeating details. According to Lemma 3 in Appendix D, we have

$$\frac{1}{NJ}\mathbb{E}\left(\|\tilde{X} - X^*\|_F^2 \mid X^*\right) \le c \min\left\{\frac{\|X^*\|_*}{J\sqrt{N}}, \frac{\|X^*\|_*^2}{NJ}, 1\right\} + ce^{-cN}, \tag{C.6}$$

Recall that we assume $\|\boldsymbol{\theta}_i^*\| \le C_0$. Following the similar arguments as in the proof of Lemma 1, we have

$$\frac{1}{NJ}\mathbb{E}\left(\|\tilde{X} - X^*\|_F^2 \mid X^*\right) \le c\frac{1}{\sqrt{J}}\left(\frac{C_0}{\delta}\right)^{\frac{K}{2}} + L\delta\left(\sqrt{C_0^2+1} + C\right) + c\exp(-cN).$$

There is a difference from the proof of Lemma 1 that the rank of matrix $f(M_\delta)$ is upper bounded by

$$\text{rank}(f(M_\delta)) \le \min\{|\mathcal{G}_1|, |\mathcal{G}_2|\} \le |\mathcal{G}_1| \le c\left(\frac{C_0}{\delta}\right)^K.$$

Choose $\delta = \left(\frac{cC_0^K}{JL^2\left(\sqrt{C_0^2+1}+C\right)^2}\right)^{\frac{1}{K+2}}$, then

$$\frac{1}{NJ}\mathbb{E}\left(\|\tilde{X} - X^*\|_F^2 \middle| X^*\right) \leq cJ^{-\frac{1}{K+2}} + c\exp\left(-cN\right).$$

Let $g(N, J) := cJ^{-\frac{1}{K+2}} + c\exp\left(-cN\right)$. By taking expectation, we have

$$\frac{1}{NJ}\mathbb{E}\left(\|\tilde{X} - X^*\|_F^2\right) \leq g(N, J).$$

For any $\Delta_{N,J} > 0$, by Chebyshev's inequality, we have

$$\Pr\left(\frac{1}{NJ}\|\tilde{X} - X^*\|_F^2 \geq \frac{g(N, J)}{\Delta_{N,J}}\right) \leq \Delta_{N,J}.$$

Thus, for any sequence $\Delta_{N,J}$ satisfying $\Delta_{N,J} = o(1)$, we have

$$\lim_{N,J\to\infty} \Pr\left(\frac{1}{NJ}\|\tilde{X} - X^*\|_F^2 \leq \frac{g(N, J)}{\Delta_{N,J}}\right) = 1.$$

In what follows, we restrict our analysis to the event $\left\{\frac{1}{NJ}\|\tilde{X} - X^*\|_F^2 \leq \frac{g(N,J)}{\Delta_{N,J}}\right\}$. By (7), we have $x_{ij}^* = f((\boldsymbol{\theta}_i^*)^\top \mathbf{a}_j^* + d_j^*) \in [2\epsilon, 1-2\epsilon]$, which leads to

$$\frac{1}{NJ}\sum_{i,j} 1_{\{\tilde{x}_{ij}\notin[\epsilon,1-\epsilon]\}} \leq \frac{g(N, J)}{\Delta_{N,J}\epsilon^2} = \frac{1}{\Delta_{N,J}\epsilon^2}\left(cJ^{-\frac{1}{K+2}} + c\exp\left(-cN\right)\right).$$

Following the similar procedure as in proof of Lemma 1, we can further bound $\|\hat{X} - X^*\|_F^2$ by

$$\begin{aligned}\frac{1}{NJ}\|\hat{X} - X^*\|_F^2 &\leq \frac{1}{\epsilon^2\Delta_{N,J}}\left(cJ^{-\frac{1}{K+2}} + c\exp(-cN)\right)\\ &= \frac{1}{\Delta_{N,J}}\left(cJ^{-\frac{1}{K+2}} + c\exp(-cN)\right). \qquad \text{(C.7)}\end{aligned}$$

To summarize, we have

$$\Pr\left(\frac{1}{NJ}\|\hat{X}-X^*\|_F^2 \le \frac{1}{\Delta_{N,J}}\left(cJ^{-\frac{1}{K+2}} + c\exp(-cN)\right)\right) \to 1, \quad \text{as } N, J \to \infty,$$

for any $\Delta_{N,J} = o(1)$. This implies $\frac{1}{NJ}\|\hat{X}-X^*\|_F^2 = O_p\left(J^{-\frac{1}{K+2}} + \exp(-cN)\right) = O_p(J^{-\frac{1}{K+2}})$, where the second equation is due to $N \ge J$. □

Proof of Proposition 4. We have

$$\begin{aligned}
L_{N,J}(A^*, \hat{A}) &= \frac{1}{JK}\min_{O\in\mathbb{R}^{K\times K}}\left\{\|A^* - \hat{A}O\|_F^2\right\} \\
&= \frac{1}{JK}\min_{O\in\mathbb{R}^{K\times K}}\left\{\|(A^*\Sigma^{\frac{1}{2}})\Sigma^{-\frac{1}{2}} - \hat{A}O\|_F^2\right\} \\
&\le \frac{1}{JK}\min_{O\in\mathbb{R}^{K\times K}}\left\{(\|A^*\Sigma^{\frac{1}{2}} - \hat{A}O\Sigma^{\frac{1}{2}}\|_F^2\right\}\|\Sigma^{-\frac{1}{2}}\|_F^2 \\
&= \frac{1}{JK}\min_{Q\in\mathbb{R}^{K\times K}}\left\{(\|A^*\Sigma^{\frac{1}{2}} - \hat{A}Q\|_F^2\right\}\|\Sigma^{-\frac{1}{2}}\|_F^2 \\
&= \frac{1}{JK}\min_{Q\in\mathbb{R}^{K\times K}}\left\{(\|\tilde{A} - \hat{A}Q\|_F^2\right\}\|\Sigma^{-\frac{1}{2}}\|_F^2 \\
&= L_{N,J}(\tilde{A}, \hat{A})\|\Sigma^{-\frac{1}{2}}\|_F^2,
\end{aligned}$$

where $\tilde{A} = A^*\Sigma^{\frac{1}{2}}$. Let $\tilde{\Theta} = (\tilde{\boldsymbol{\theta}}_1, ..., \tilde{\boldsymbol{\theta}}_N)^\top = \Theta^*\Sigma^{-\frac{1}{2}}$. Then $\Theta^*(A^*)^\top = \tilde{\Theta}\tilde{A}^\top$, and $\tilde{\boldsymbol{\theta}}_i$s are independent and identically distributed from a distribution $\tilde{F}$ which has mean $\mathbf{0}$ and covariance matrix I_K. Therefore, it suffices to show $L_{N,J}(A^*, \hat{A}) \overset{pr}{\to} 0$ when $\Sigma = I_K$. We prove it through the following two lemmas whose proofs are given in Appendix D.

Lemma 1. *Assume conditions A1, A2, A3, A5 and A6 are satisfied and further assume that* (3) *and* (4) *are satisfied. Then,*

$$\frac{1}{NJ}\left\|\hat{\Theta}\hat{A}^\top - \Theta^*(A^*)^\top\right\|_F^2 \overset{pr}{\to} 0.,$$

where $\hat{\Theta}$ and $\hat{A}$ are given in Algorithm 2.

Lemma 2. *Suppose conditions A1, A2 and A4 are satisfied and further suppose that*

$$\frac{1}{NJ}\left\|\hat{\Theta}\hat{A}^{\top}-\Theta^{*}(A^{*})^{\top}\right\|_{F}^{2}\overset{pr}{\to}0.$$

Then, $L_{N,J}(A^{*},\hat{A})\overset{pr}{\to}0$.

We complete the proof. □

D Proof of Lemmas

Proof of Lemma 1. We first give a lemma regarding the error bound for recovering the probability matrix X^{*}.

Lemma 3. *Given* X^{*}*, we have*

$$\frac{1}{NJ}\mathbb{E}\left(\|\tilde{X}-X^{*}\|_{F}^{2}\Big|X^{*}\right)\leq c\min\left\{\frac{\|X^{*}\|_{*}}{J\sqrt{N}},\frac{\|X^{*}\|_{*}^{2}}{NJ},1\right\}+ce^{-cN}. \tag{D.1}$$

Let

$$p_{\epsilon}:=\Pr(\|\boldsymbol{\theta}_{1}^{*}\|>C_{\epsilon}),$$

where $C_{\epsilon}=h(2\epsilon)/C$ is a quantity depending on ϵ. Let

$$\mathcal{A}_{N,J}:=\left\{\|\boldsymbol{\theta}_{i}^{*}\|\leq C_{\epsilon},\ \text{for } i=1,...,N\right\}.$$

Then, according to the condition (3)

$$\lim_{N,J\to\infty}\Pr(\mathcal{A}_{N,J})=\lim_{N,J\to\infty}(1-p_{\epsilon})^{N}=1.$$

In what follows, we restrict the analysis to the event $\mathcal{A}_{N,J}$. Let $\mathcal{G}_{1},\mathcal{G}_{2}$ be two δ-nets for

$B_0^{(K)}(C_\epsilon)$ and $B_0^{(K+1)}(C)$, respectively. This means $\mathcal{G}_1 \subset B_0^{(K)}(C_\epsilon), \mathcal{G}_2 \subset B_0^{(K+1)}(C)$ and

$$B_0^{(K)}(C_\epsilon) \subset \bigcup_{\mathbf{x}\in\mathcal{G}_1} B_{\mathbf{x}}^{(K)}(\delta), \quad B_0^{(K+1)}(C) \subset \bigcup_{\mathbf{x}\in\mathcal{G}_2} B_{\mathbf{x}}^{(K+1)}(\delta).$$

For any $\boldsymbol{\theta}_i^*$, let $p(\boldsymbol{\theta}_i^*)$ be a point in $\mathcal{G}_1$ such that

$$\|\boldsymbol{\theta}_i^* - p(\boldsymbol{\theta}_i^*)\| \leq \delta,$$

which implies

$$\|\boldsymbol{\theta}_i^+ - (1, p(\boldsymbol{\theta}_i^*)^\top)^\top\| = \|\boldsymbol{\theta}_i^* - p(\boldsymbol{\theta}_i^*)\| \leq \delta.$$

With a little abuse of notation, we use $p(\boldsymbol{\theta}_i^+)$ to denote $(1, p(\boldsymbol{\theta}_i^*)^\top)^\top$. For any $\mathbf{a}_j^+$, let $p(\mathbf{a}_j^+)$ be a point in $\mathcal{G}_2$ such that

$$\|\mathbf{a}_j^+ - p(\mathbf{a}_j^+)\| \leq \delta.$$

It is not hard to see that we can find such $\mathcal{G}_1, \mathcal{G}_2$ such that

$$|\mathcal{G}_1| \leq c\left(\frac{C_\epsilon}{\delta}\right)^K, \quad |\mathcal{G}_2| \leq c\left(\frac{C}{\delta}\right)^{K+1},$$

This is due to definition of $\mathcal{A}_{N,J}$ and condition A1. Let $M_\delta = ({}_\delta m_{ij})_{N\times J}$, where ${}_\delta m_{ij} = f\left(p(\boldsymbol{\theta}_i^+)^\top p(\mathbf{a}_j^+)\right)$, then we have

$$\text{rank}(M_\delta) \leq \min\{|\mathcal{G}_1|, |\mathcal{G}_2|\} \leq |\mathcal{G}_2| \leq c\left(\frac{C}{\delta}\right)^{K+1}.$$

Now we provide an upper bound for $\|X^*\|_*$ on the right-hand side of (D.1). We have

$$\|X^*\|_* = \|f(M^*)\|_* \leq \overbrace{\|f(M^*) - f(M_\delta)\|_*}^{(I)} + \overbrace{\|f(M_\delta)\|_*}^{(II)}. \tag{D.2}$$

The second term on the right-hand side of the above display is bounded above by

$$(II) \le \sqrt{\mathrm{rank}(f(M_\delta))} \cdot \|f(M_\delta)\|_F \le c \left(\frac{C}{\delta}\right)^{\frac{K+1}{2}} \sqrt{NJ}. \tag{D.3}$$

Now we consider the first term. We have

$$\begin{aligned} \left|(\boldsymbol{\theta}_i^+)^\top \mathbf{a}_j^+ - (p(\boldsymbol{\theta}_i^+))^\top p(\mathbf{a}_j^+)\right| &\le \left|(\boldsymbol{\theta}_i^+)^\top (\mathbf{a}_j^+ - p(\mathbf{a}_j^+))\right| + \left|(\boldsymbol{\theta}_i^+ - p(\boldsymbol{\theta}_i^+))^\top p(\mathbf{a}_j^+)\right| \\ &\le \sqrt{C_\epsilon^2 + 1} \cdot \delta + \delta C. \end{aligned}$$

So

$$\begin{aligned} |f(m_{ij}^*) - f({}_\delta m_{ij})| &= \left|f\left((\boldsymbol{\theta}_i^+)^\top \mathbf{a}_j^+\right) - f((p(\boldsymbol{\theta}_i^+))^\top p(\mathbf{a}_j^+))\right| \\ &\le L\delta\left(\sqrt{C_\epsilon^2 + 1} + C\right). \end{aligned}$$

We have used the Lipschitz continuity in condition A3 here. Then the first term in (D.2) is bounded from above as

$$(I) \le \sqrt{J}\|f(M^*) - f(M_\delta)\|_F \le L\delta\left(\sqrt{C_\epsilon^2 + 1} + C\right)\sqrt{J}\sqrt{NJ}. \tag{D.4}$$

Here we used the fact that the rank of the matrix $f(M^*) - f(M_\delta)$ cannot exceed J according to condition A5. Combined (D.1), (D.2), (D.3) and (D.4), then on the event $\mathcal{A}_{N,J}$,

$$\frac{1}{NJ}\mathbb{E}\left(\|\tilde{X} - X^*\|_F^2 \middle| X^*\right) \le c\frac{1}{\sqrt{J}}\left(\frac{C}{\delta}\right)^{\frac{K+1}{2}} + L\delta\left(\sqrt{C_\epsilon^2 + 1} + C\right) + c\exp(-cN).$$

Choose $\delta = \left(\frac{cC^{K+1}}{JL^2(\sqrt{C_\epsilon^2+1}+C)^2}\right)^{\frac{1}{K+3}}$, then

$$\frac{1}{NJ}\mathbb{E}\left(\|\tilde{X} - X^*\|_F^2 \middle| X^*\right) \le cC_\epsilon^{\frac{K+1}{K+3}} J^{\frac{-1}{K+3}} + c\exp(-cN),$$

which implies

$$\frac{1}{NJ}\mathbb{E}\left(\|\tilde{X}-X^*\|_F^2 \mid \mathcal{A}_{N,J}\right) \leq g(N,J),$$

where we define $g(N,J) := cC_\epsilon^{\frac{K+1}{K+3}}J^{\frac{-1}{K+3}} + c\exp(-cN)$. By Chebyshev's inequality, for any $\Delta_{N,J} > 0$,

$$\Pr\left(\frac{1}{NJ}\|\tilde{X}-X^*\|_F^2 \geq \frac{g(N,J)}{\Delta_{N,J}}\Big|\mathcal{A}_{N,J}\right) \leq \Delta_{N,J}.$$

Thus,

$$\Pr\left(\frac{1}{NJ}\|\tilde{X}-X^*\|_F^2 \leq \frac{g(N,J)}{\Delta_{N,J}}\Big|\mathcal{A}_{N,J}\right) \geq 1-\Delta_{N,J}. \tag{D.5}$$

Let $\mathcal{B}_{N,J} := \mathcal{A}_{N,J} \cap \{\frac{1}{NJ}\|\tilde{X}-X^*\|_F^2 \leq \frac{g(N,J)}{\Delta_{N,J}}\}$, then according to (D.5) for any sequence $\Delta_{N,J}$ satisfying $\Delta_{N,J} = o(1)$, we have

$$\lim_{N,J\to\infty}\Pr\left(\mathcal{B}_{N,J}\right) = \lim_{N,J\to\infty}\Pr\left(\mathcal{A}_{N,J}\right)\cdot\lim_{N,J\to\infty}\Pr\left(\frac{1}{NJ}\|\tilde{X}-X^*\|_F^2 \leq \frac{g(N,J)}{\Delta_{N,J}}\Big|\mathcal{A}_{N,J}\right) = 1.$$

We will restrict our analysis on $\mathcal{B}_{N,J}$ in what follows. Let $h(N,J) = \frac{g(N,J)}{\Delta_{N,J}}$, then on $\mathcal{B}_{N,J}$, we have $\frac{1}{NJ}\|\tilde{X}-X^*\|_F^2 \leq h(N,J)$.

Recall $C_\epsilon = \frac{h(2\epsilon)}{C}$. Then, according to the definition of the function h and C_ϵ, we can see that $f(CC_\epsilon), f(-CC_\epsilon) \in [2\epsilon, 1-2\epsilon]$. This interval is non-empty because $\epsilon \leq \frac{1}{4}$. Thus, when the event $\mathcal{B}_{N,J}$ happens, we have $x_{ij}^* = f((\boldsymbol{\theta}_i^+)^\top \mathbf{a}_j^+) \in [2\epsilon, 1-2\epsilon]$, which leads to

$$\frac{1}{NJ}\sum_{i,j}1_{\{\tilde{x}_{ij}\notin[\epsilon,1-\epsilon]\}} \leq \frac{1}{NJ}\sum_{i,j}1_{\{|\tilde{x}_{ij}-x_{ij}^*|\geq\epsilon\}} \leq \frac{1}{NJ}\sum_{i,j}\frac{(\tilde{x}_{ij}-x_{ij}^*)^2}{\epsilon^2} \leq \frac{h(N,J)}{\epsilon^2}.$$

Since $\hat{X}$ and $\tilde{X}$ are not far away from each other by definition, we can bound $\|\hat{X} - X^*\|_F^2$ by

$$\begin{aligned}
\frac{1}{NJ}\|\hat{X} - X^*\|_F^2 &= \frac{1}{NJ}\sum_{i,j}\left[(\tilde{x}_{ij} - x_{ij}^*)^2 1_{\{\tilde{x}_{ij}\in[\epsilon,1-\epsilon]\}} + (\hat{x}_{ij} - x_{ij}^*)^2 1_{\{\tilde{x}_{ij}\notin[\epsilon,1-\epsilon]\}}\right] \\
&\leq \frac{1}{NJ}\sum_{i,j}(\tilde{x}_{ij} - x_{ij}^*)^2 + \frac{1}{NJ}\sum_{i,j}(1-3\epsilon)^2\, 1_{\{\tilde{x}_{ij}\notin[\epsilon,1-\epsilon]\}} \\
&\leq \left(1 + \left(\frac{1-3\epsilon}{\epsilon}\right)^2\right) h(N,J) \\
&\leq \frac{1}{\epsilon^2} h(N,J) \qquad \text{(D.6)}
\end{aligned}$$

where the last inequality is because $\epsilon \leq \frac{1}{4}$. According to condition A3 and the above inequality, we have

$$\begin{aligned}
\frac{1}{NJ}\|\tilde{M} - M^*\|_F^2 &= \frac{1}{NJ}\|f^{-1}(\hat{X}) - f^{-1}(X^*)\|_F^2 \qquad \text{(D.7)} \\
&\leq \frac{1}{(g(\epsilon))^2}\frac{1}{NJ}\|\hat{X} - X^*\|_F^2 \qquad \text{(D.8)} \\
&\leq \frac{1}{(\epsilon g(\epsilon))^2} h(N,J). \qquad \text{(D.9)}
\end{aligned}$$

The first inequality holds because $x_{ij}^*, \hat{x}_{ij} \in [\epsilon, 1-\epsilon]$ on the event $\mathcal{B}_{N,J}$.

We proceed to an upper bound of $\hat{M} - \Theta^*(A^*)^\top$. Recall that $M^* = \mathbf{1}_N(d^*)^\top + \Theta^*(A^*)^\top$, $\tilde{M} = \hat{M} + \mathbf{1}_N\hat{d}$. Let $H_1 = \hat{M} - \Theta^*(A^*)^\top$ and $H_2 = \mathbf{1}_N(\hat{d})^\top - \mathbf{1}_N(d^*)^\top$. We have

$$\frac{1}{NJ}\|H_1 + H_2\|_F^2 = \frac{1}{NJ}\left(\|H_1\|_F^2 + \|H_2\|_F^2 + 2tr\{H_1^\top H_2\}\right). \qquad \text{(D.10)}$$

We first bound the trace term in the above display,

$$
\begin{aligned}
\left|tr\{H_1^\top H_2\}\right| &= \left|tr\{(A^*(\Theta^*)^\top - \hat{M}^\top)\mathbf{1}_N(\hat{\mathbf{d}}-\mathbf{d}^*)^\top\}\right| \\
&= \left|tr\{A^*(\Theta^*)^\top\mathbf{1}_N(\hat{\mathbf{d}}-\mathbf{d}^*)^\top\}\right|, \quad (\hat{M}^\top\mathbf{1}_N = \mathbf{0}_J) \\
&= \left|(\hat{\mathbf{d}}-\mathbf{d}^*)^\top A^*(\Theta^*)^\top\mathbf{1}_N\right|, \quad \text{(exchangeability for trace operator)} \\
&= \left|\left\langle \sum_j (\hat{d}_j - d_j^*)\mathbf{a}_j^*, \sum_i \boldsymbol{\theta}_i^* \right\rangle\right| \\
&\le \left\|\sum_j (\hat{d}_j - d_j^*)\mathbf{a}_j^*\right\| \left\|\sum_i \boldsymbol{\theta}_i^*\right\|. \quad \text{(Cauchy-Schwarz inequality)}
\end{aligned}
$$

Through simple algebra, we have $d_j^* = \frac{1}{N}\sum_{i=1}^N \left(m_{ij}^* + (\boldsymbol{\theta}_i^*)^\top\mathbf{a}_j^*\right)$. By the definition of $\hat{d}_j$, we have $\hat{d}_j = \frac{1}{N}\sum_{i=1}^N \tilde{m}_{ij}$. Then

$$
\begin{aligned}
|\hat{d}_j - d_j^*| &\le \left|\frac{1}{N}\sum_i (\tilde{m}_{ij} - m_{ij}^*)\right| + \left|\frac{1}{N}\sum_i (\boldsymbol{\theta}_i^*)^\top\mathbf{a}_j^*\right| \\
&\le \left|\frac{1}{N}\sum_i (\tilde{m}_{ij} - m_{ij}^*)\right| + \left\|\frac{1}{N}\sum_i \boldsymbol{\theta}_i^*\right\| \left\|\mathbf{a}_j^*\right\|,
\end{aligned}
$$

which leads to

$$
\begin{aligned}
\left\|\sum_j (\hat{d}_j - d_j^*)\mathbf{a}_j^*\right\| &\le \sum_j |\hat{d}_j - d_j^*|\|\mathbf{a}_j^*\| \\
&\le C\sum_j |\hat{d}_j - d_j^*|, \quad (\|\mathbf{a}_j^*\| \le C) \\
&\le C\sum_j \left\{\left|\frac{1}{N}\sum_i (\tilde{m}_{ij} - m_{ij}^*)\right| + \left\|\frac{1}{N}\sum_i \boldsymbol{\theta}_i^*\right\| \left\|\mathbf{a}_j^*\right\|\right\} \\
&\le \frac{C}{N}\sum_{i,j} |\tilde{m}_{ij} - m_{ij}^*| + C^2 J\left\|\frac{1}{N}\sum_i \boldsymbol{\theta}_i^*\right\|, \quad (\|\mathbf{a}_j^*\| \le C) \\
&\le CJ\sqrt{\frac{1}{NJ}\|\tilde{M} - M^*\|_F^2} + C^2 J\left\|\frac{1}{N}\sum_i \boldsymbol{\theta}_i^*\right\|. \quad \text{(Cauchy-Schwarz inequality)}
\end{aligned}
$$

So we can bound $\left|tr\{H_1^\top H_2\}\right|$ by

$$\left|tr\{H_1^\top H_2\}\right| \le \left(CJ\sqrt{\frac{1}{NJ}\|\tilde{M} - M^*\|_F^2} + C^2 J \left\| \frac{1}{N}\sum_i \boldsymbol{\theta}_i^* \right\| \right) \left\| \sum_i \boldsymbol{\theta}_i^* \right\| \tag{D.11}$$

According to condition A2 and law of large number, we have

$$\Pr\left(\frac{1}{N}\left\| \sum_{i=1}^N \boldsymbol{\theta}_i^* \right\| \le \xi \right) \to 1, \quad \text{as } N, J \to \infty,$$

for any $\xi > 0$. Let

$$\mathcal{C}_{N,J,\xi} := \left\{ \frac{1}{N}\left\| \sum_{i=1}^N \boldsymbol{\theta}_i^* \right\| \le \xi \right\} \cap \mathcal{B}_{N,J},$$

then we have

$$\Pr(\mathcal{C}_{N,J,\xi}) \to 1, \quad \text{as } N, J \to \infty,$$

for any $\xi > 0$. On $\mathcal{C}_{N,J,\xi}$, according to (D.7) , (D.10) and (D.11),

$$\begin{aligned} \frac{1}{NJ}\|\Theta^*(A^*)^\top - \hat{M}\|_F^2 = \frac{1}{NJ}\|H_1\|_F^2 \le \frac{1}{NJ}\|\tilde{M} - M^*\|_F^2 + \frac{2}{NJ}\left|tr\{H_1^\top H_2\}\right| \\ \le \frac{h(N,J)}{(\epsilon g(\epsilon))^2} + C\xi\left(\frac{\sqrt{h(N,J)}}{\epsilon g(\epsilon)} + C\xi \right). \end{aligned} \tag{D.12}$$

Recall how we get $\hat{\Theta}, \hat{A}$ in algorithm 2 and we have

$$
\begin{aligned}
&\|\hat{M} - \hat{\Theta}\hat{A}^\top\|_2 \\
=&\sigma_{K+1}(\hat{M}) \\
=&|\sigma_{K+1}(\hat{M}) - \sigma_{K+1}(\Theta^*(A^*)^\top)| \\
\leq&\|\hat{M} - \Theta^*(A^*)^\top\|_2 \\
\leq&\|\Theta^*(A^*)^\top - \hat{M}\|_F.
\end{aligned}
$$

So

$$
\|\hat{\Theta}\hat{A}^\top - \Theta^*(A^*)^\top\|_2 \leq \|\hat{\Theta}\hat{A}^\top - \hat{M}\|_2 + \|\hat{M} - \Theta^*(A^*)^\top\|_2 \leq 2\|\hat{M} - \Theta^*(A^*)^\top\|_F, \tag{D.13}
$$

which leads to

$$
\begin{aligned}
\frac{1}{NJ}\|\hat{\Theta}\hat{A}^\top - \Theta^*(A^*)^\top\|_F^2 &\leq \frac{2K}{NJ}\|\hat{\Theta}\hat{A}^\top - \Theta^*(A^*)^\top\|_2^2, \\
&\leq \frac{8K}{NJ}\|\hat{M} - \Theta^*(A^*)^\top\|_F^2, \\
&\leq 8K\frac{h(N,J)}{(\epsilon g(\epsilon))^2} + 8KC\xi\left(\frac{\sqrt{h(N,J)}}{\epsilon g(\epsilon)} + C\xi\right), \qquad (D.14)
\end{aligned}
$$

where the first inequality is due to $\text{rank}\big(\hat{\Theta}\hat{A}^\top - \Theta^*(A^*)^\top\big) \leq 2K$, the second inequality is due to (D.13) and the last inequality is due to (D.12). Thus, on the event $\mathcal{C}_{N,J,\xi}$

$$
\frac{1}{NJ}\|\hat{\Theta}\hat{A}^\top - \Theta^*(A^*)^\top\|_F^2 = O\left(\frac{h(N,J)}{(\epsilon g(\epsilon))^2} + \xi\left(\frac{\sqrt{h(N,J)}}{\epsilon g(\epsilon)} + \xi\right)\right).
$$

Recall

$$
\frac{h(N,J)}{(\epsilon g(\epsilon))^2} = \frac{c}{\Delta_{N,J}}\left(\frac{(h(2\epsilon))^{\frac{K+1}{K+3}}}{(\epsilon g(\epsilon))^2 J^{\frac{1}{K+3}}} + \frac{\exp(-cN)}{(\epsilon g(\epsilon))^2}\right),
$$

where $\Delta_{N,J}$ could be any sequence satisfying $\Delta_{N,J} = o(1)$. By (3), (4) and condition A5, there exists $\Delta_{N,J} = o(1)$ such that $\frac{h(N,J)}{(\epsilon g(\epsilon))^2} = o(1)$. So fix any $\xi < 1$, for N, J large enough, we have $\frac{h(N,J)}{(\epsilon g(\epsilon))^2} \leq \xi$. Then there is a constant κ such that for N, J large enough, on $C_{N,J,\xi}$ with $\xi \in (0,1)$, we have,

$$\frac{1}{NJ}\|\hat{\Theta}\hat{A}^\top - \Theta^*(A^*)^\top\|_F^2 \leq \kappa\xi. \tag{D.15}$$

This combined with $\Pr(C_{N,J,\xi}) \to 1$ for any ξ sufficiently small completes the proof. □

Proof of Lemma 2. Let

$$Q^{(N,J)} = \frac{1}{\sqrt{N}}\hat{\Theta}^\top\Theta^*\left((\Theta^*)^\top\Theta^*\right)^{-\frac{1}{2}}$$

and in the following we will show that

$$\frac{1}{JK}\|A^* - \hat{A}Q^{(N,J)}\|_F^2 \overset{pr}{\to} 0.$$

For any $\alpha > 0$, let

$$\mathcal{D}_{N,J,\alpha} := \left\{1 - \alpha \leq \frac{\sigma_K(\Theta^*)}{\sqrt{N}} \leq \frac{\sigma_1(\Theta^*)}{\sqrt{N}} \leq 1 + \alpha\right\}. \tag{D.16}$$

Applying Theorem 5.39 of Vershynin (2010) to the matrix Θ^*, we have $\lim_{N,J\to\infty}\Pr(\mathcal{D}_{N,J,\alpha}) = 1$ for any $\alpha > 0$. We restrict our analysis on $\mathcal{D}_{N,J,\alpha}$ in what follows and denote

$$Q(N,J) := \frac{1}{NJ}\|\hat{\Theta}\hat{A}^\top - \Theta^*(A^*)^\top\|_F^2.$$

Then,

$$\begin{aligned}\|A^* - \hat{A}Q^{(N,J)}\|_F =& \|A^* - \hat{A}\frac{1}{\sqrt{N}}\hat{\Theta}^\top\Theta^*\left((\Theta^*)^\top\Theta^*\right)^{-\frac{1}{2}}\|_F \\ \leq& \underbrace{\|A^* - A^*\frac{1}{\sqrt{N}}\left((\Theta^*)^\top\Theta^*\right)^{\frac{1}{2}}\|_F}_{(a)} + \underbrace{\|(A^*(\Theta^*)^\top - \hat{A}\hat{\Theta}^\top)\frac{1}{\sqrt{N}}\Theta^*((\Theta^*)^\top\Theta^*)^{-\frac{1}{2}}\|_F}_{(b)}.\end{aligned} \tag{D.17}$$

We consider (b) first:

$$\begin{aligned}(b) &\le \|A^*(\Theta^*)^\top - \hat{A}\hat{\Theta}^\top\|_F \frac{1}{\sqrt{N}}\|\Theta^*\|_2\|((\Theta^*)^\top\Theta^*)^{-\frac{1}{2}}\|_2 \\ &= \sqrt{NJ}\sqrt{Q(N,J)}\frac{\sigma_1(\Theta^*)}{\sqrt{N}}\frac{1}{\sigma_K(\Theta^*)} \\ &\le \sqrt{JQ(N,J)}\frac{1+\alpha}{1-\alpha}, \quad \text{(by (D.16))}\end{aligned} \tag{D.18}$$

For (a), notice that

$$\begin{aligned}\left\|\frac{1}{\sqrt{N}}\left((\Theta^*)^\top\Theta^*\right)^{\frac{1}{2}} - I_K\right\|_2 &= \max_{1\le k\le K}\left|\frac{\sigma_k(\Theta^*)}{\sqrt{N}} - 1\right| \\ &\le \alpha. \quad \text{(by (D.16))}\end{aligned}$$

So

$$(a) \le \|A^*\|_F \left\|\frac{1}{\sqrt{N}}\left((\Theta^*)^\top\Theta^*\right)^{\frac{1}{2}} - I_K\right\|_2 \le C\sqrt{J}\alpha. \tag{D.19}$$

Combine (D.17), (D.18) and (D.19), we get on $\mathcal{D}_{N,J,\alpha}$

$$\frac{1}{\sqrt{JK}}\|A^* - \hat{A}Q\|_F \le \frac{C\alpha}{\sqrt{K}} + \frac{1+\alpha}{\sqrt{K}(1-\alpha)}\sqrt{Q(N,J)}.$$

Recall that $Q(N,J) = \frac{1}{NJ}\|\hat{\Theta}\hat{A}^\top - \Theta^*(A^*)^\top\|_F^2 \xrightarrow{pr} 0$, α can be arbitrarily small and $\Pr(\mathcal{D}_{N,J,\alpha}) \to 1$, we complete the proof. □

Proof of Lemma 3. This lemma is almost the same as Theorem 1.1 of Chatterjee (2015) by setting, in his notations, $\eta = 0.02$ and $\sigma^2 = 1/4$, except two small differences. The first is that the probability p can be changed through N, J in the setting of Chatterjee (2015) while p is a constant in our setting. Therefore we absorb p into constants c in the LHS of (D.1). The second difference is a modification in step 5 of Algorithm 2 that we require X to include at least $K+1$ singular values of Z. This does not change the result of Theorem 1.1

of Chatterjee (2015) given the following lemma which is based on Lemma 3.5 of Chatterjee (2015).

Lemma 4. *For fixed $0 < m \leq n$ and a $m \times n$ matrix A, let $A = \sum_{i=1}^m \sigma_i x_i y_i^\top$ be the singular value decomposition of A. Fix any $\delta > 0$ and integer $T > 0$, and define*

$$\tilde{B} := \sum_{i=1}^{l} \sigma_i x_i y_i^\top,$$

where $l = \max\{T, \arg\max\{i : \sigma_i > (1+\delta)\|A - B\|\}\}$. Then

$$\|\tilde{B} - B\|_F \leq (1+\delta)\sqrt{T}\|A - B\| + K(\delta)\left(\|A - B\|\|B\|_*\right)^{\frac{1}{2}}, \tag{D.20}$$

where $K(\delta) = (4 + 2\delta)\sqrt{2/\delta} + \sqrt{2+\delta}$.

Notice that we have another term $(1+\delta)\sqrt{T}\|A - B\|$ in (D.20) compared with Lemma 3.5 in Chatterjee (2015), which is due to the composition of $\tilde{B}$. In the proof of Theorem 1.1 in Chatterjee (2015), by replacing Lemma 3.5 in Chatterjee (2015) by the above lemma with $T = K + 1$, we get

$$\frac{1}{NJ}\mathbb{E}\left(\|\tilde{X} - X^*\|_F^2 \middle| X^*\right) \leq c\min\left\{\frac{\|X^*\|_*}{J\sqrt{N}} + \frac{1}{J}, \frac{\|X^*\|_*^2}{NJ}, 1\right\} + ce^{-cN}. \tag{D.21}$$

The $1/J$ term in (D.21) results from the first term in (D.20). Notice that if

$$\frac{\|X^*\|_*}{J\sqrt{N}} + \frac{1}{J} \leq \frac{\|X^*\|_*^2}{NJ},$$

then

$$\frac{\|X^*\|_*}{J\sqrt{N}} \leq \frac{\|X^*\|_*^2}{NJ},$$

which leads to

$$\frac{\|X^*\|_*}{J\sqrt{N}} \geq \frac{1}{J}.$$

Therefore we can remove the $1/J$ term in (D.21) to complete the proof. $\square$

Proof of Lemma 4. Let

$$\hat{B} := \sum_{i:\sigma_i > (1+\delta)\|A-B\|} \sigma_i x_i y_i^\top$$

and by Lemma 3.5 of Chatterjee (2015), we have

$$\|\tilde{B} - B\|_F \leq K(\delta) \left(\|A - B\| \|B\|_*\right)^{\frac{1}{2}}.$$

Note that

$$\|\tilde{B} - \hat{B}\|_F \leq \sqrt{T}(1+\delta)\|A - B\|$$

and we complete the proof by triangular inequality. $\square$

References

Chatterjee, S. (2015). Matrix estimation by universal singular value thresholding. *The Annals of Statistics*, 43:177–214.

Vershynin, R. (2010). Introduction to the non-asymptotic analysis of random matrices. *arXiv preprint arXiv:1011.3027*.
